# Error Analysis and Propagation in Metabolomics Data Analysis

**DOI:** 10.5936/csbj.201301006

**Published:** 2013-02-19

**Authors:** Hunter N.B. Moseley

**Affiliations:** a Department of Chemistry, Center for Regulatory and Environmental Analytical Metabolomics, University of Louisville, Louisville, Kentucky, USA

**Keywords:** metabolomics, error analysis, error propagation, nuclear magnetic resonance, mass spectrometry

## Abstract

Error analysis plays a fundamental role in describing the uncertainty in experimental results. It has several fundamental uses in metabolomics including experimental design, quality control of experiments, the selection of appropriate statistical methods, and the determination of uncertainty in results. Furthermore, the importance of error analysis has grown with the increasing number, complexity, and heterogeneity of measurements characteristic of ‘omics research. The increase in data complexity is particularly problematic for metabolomics, which has more heterogeneity than other omics technologies due to the much wider range of molecular entities detected and measured. This review introduces the fundamental concepts of error analysis as they apply to a wide range of metabolomics experimental designs and it discusses current methodologies for determining the propagation of uncertainty in appropriate metabolomics data analysis. These methodologies include analytical derivation and approximation techniques, Monte Carlo error analysis, and error analysis in metabolic inverse problems. Current limitations of each methodology with respect to metabolomics data analysis are also discussed.

## Introduction

Error analysis is the detection, identification, and quantification of different types of uncertainty present in measurements and the propagation of this uncertainty through mathematical calculations and procedures. This definition associates the term *error* more with precision and less with mistake or accuracy. As such, error analysis plays a fundamental role in describing the degree of confidence in results derived from experiments across a variety of disciplines. Naturally, the importance of error analysis has grown with the increase in the number and heterogeneity of measurements obtained from newer, high-throughput, omics-level technologies.

The increase in heterogeneity is particularly problematic for metabolomics, which has more heterogeneity than other omics technologies due to the much wider range of molecular entities detected and measured: thousands of distinct metabolites versus single classes of repetitive linear polymers like DNA, RNA, and proteins. Also within the context of metabolomics, error analysis has several fundamental uses: i) the improvement of experimental design, ii) the quality control of experiments, iii) the selection of appropriate statistical methods, and iv) the determination of uncertainty in results. This review will introduce the fundamental concepts of error analysis as they apply to a wide range of metabolomics experimental designs and will discuss current methodologies for determining the propagation of uncertainty in appropriate metabolomics data analysis, with increasing level of statistical detail as the review progresses.

### Basic statistical terminology

Owing to the confusion and misconceptions about statistical definitions within published metabolomics literature, I begin by concisely defining the main statistical terminology and concepts used throughout the rest of this review, but this paper is no substitute for more in-depth reading and study [1–3]. As shown in Table 1, the estimate of an expected value is usually represented as the mean or average of repeated measured values ($\bar{x}$) of a given measured variable x. The median, defined as the middle value in a sorted list of repeated measured values, is another common estimate of an expected value, often preferred when the distribution of the measured variable is significantly skewed (non-symmetric). The variance $\sigma_{x}^{2}$ represents the spread of these repeated measured values around this mean. The standard deviation σ_x_ is just the square root of the variance. Another useful term for describing uncertainty is the standard error $\sigma_{\bar{x}}$ (also known as the standard deviation of the mean), which is a probabilistic description of how close the mean is to the expected value.

**Table 1:** Common statistical terms and their mathematical definitions.

Term	Equation
Mean (estimate of the expected value)	$\bar{x}=\frac{\sum_{i}^{N}x_{i}}{N}$
Variance	$\sigma_{x}^{2}=\frac{\sum_{i}^{N}{\left(x_{i}-\bar{x}\right)}^{2}}{N-1}$
Standard Error	$SE_{x}or\sigma_{\bar{x}}=\frac{\sigma_{x}}{\sqrt{N}}$
Covariance	$\sigma_{xy}^{2}=\frac{\sum_{i}^{N}(x_{i}-\bar{x})(y_{i}-\bar{y})}{N-1}$
a(Pearson's) Correlation	$r_{xy}=\frac{\sum_{i}^{N}(x_{i}-\bar{x})(y_{i}-\bar{y})}{(N-1)\sigma_{x}\sigma_{y}}$

a Pearson's correlation coefficient [4]

A related term is a confidence interval, which identifies a range that includes the expected value at some level of confidence (typically 95% or 99%). A multidimensional generalization of a confidence interval is a confidence region, typically approximated with an elliptical shape. Both confidence intervals and confidence regions are especially useful descriptions of uncertainty when the probability distribution of the measurement(s) is unknown and clearly non-normal. However, the term confidence interval should not be confused with a tolerance interval which is more analogous to standard deviation and describes a range that includes a certain proportion of the population. Next, covariance $\sigma_{\mathrm{xy}}^{2}$ describes how two measured variables vary together. And correlation r_xy_ describes the dependence between two measured variables. Both of these terms are useful in describing the relationship between measured variables.

Finally, the term statistical power is the probability that a statistical test will properly reject the null hypothesis and not make a false negative decision (Type II error). A null hypothesis is a falsifiable assertion. A statistical test is a method for making decisions from data by deciding whether to reject a null hypothesis at a certain level of significance. Statistical methods can be divided into parametric methods that assume an underlying probability distribution for the data and non-parametric methods that make no such assumptions and treats data from a categorical or ordinal (having an order or ranking) perspective. A p-value is the probability that a true null hypothesis would have an observed value at a certain extreme or worse. And a Type I error is the improper rejection of a null hypothesis, also known as a false positive.

### Types of variance and error

**Figure 1:**
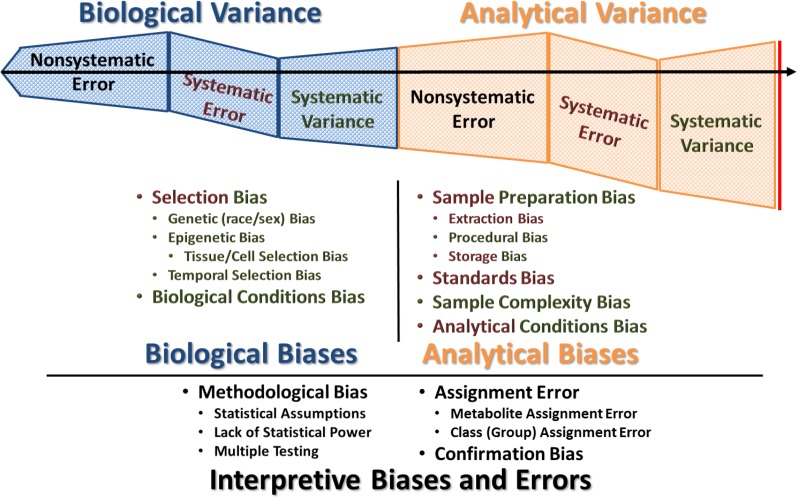
**The major divisions of variance, error, and bias in bioanalytical experiments.** The expanding cone represents the effects of major types of variance and error on the spread (red line) and distribution of measured values. The major divisions of variance by source are biological versus analytical variance. The major divisions of error are systematic versus nonsystematic error, with a third related entity, systematic variance, representing the variance between groups of related samples in the sample set. The major divisions of bias are biological, analytical, and interpretive, based upon when their effects manifest in the bioanalytical experiment and data analysis.

The major divisions of variance in bioanalytical experiments are biological versus analytical variance, which categorize the source of the variance (Figure 1). Biological variance arises from the spread of measured values observed from multiple biological samples due to differences in individuals. Analytical variance arises from the spread of measured values observed from multiple measurements made from the same biological sample, including all technical steps from sample acquisition to primary analytical data collection. More often, the biological variance is significantly larger than the analytical variance. The major divisions of error are systematic versus nonsystematic (random) error, which describe the type of error. Systematic error is a type of uncertainty not revealed by repeated measurements and represents biases in measurements that must be tested for separately in order to address or correct. While systematic error does not appreciably affect variance, it can affect covariances and correlations between measured variables. Nonsystematic error, also known as error variance, is the experimental uncertainty revealed by repeated measurements and can be reliably estimated by statistical methods.

A third kind of variance is systematic variance, which represents the variance between groups of related samples in the sample set. Depending on the specific experiment and the applied statistical analysis, specific systematic variances can be part of the detectable signal or part of the uncertainty in the measurements due to confounding factors. In other words, one scientist's uncertainty is another scientist's usable systematic variance. For example, in matched case-control measurements, the intergroup variance is the actual desired signal to detect. In other experiments, differences in group composition between case-control measurements like subject sex (male/female) can be an additional source of confounding systematic variance. In the special case when the sample set is unintentionally uniform (i.e. homogeneous), the group effect on the measurements will be systematic error and will not contribute to systematic variance. The classification of systematic error is further complicated by interpretive errors that often have the appearance of systematic error. The classic example is the error introduced by a “standard” measurement used in the correction of other measurements. Also, these divisions in variance and error are not mutually exclusive (Figure 1). Both biological and analytical variance can be divided into systematic variance and nonsystematic error components.

### Typical biases in metabolomics

Most forms of error and confounding variance in measured variables originate from some type of bias. This term *bias* refers to any factor that distorts the design, execution, analysis, and interpretation of a measurement [5] and is an ever-present problem for metabolomics experiments [6, 7]. A bias typically causes: i) a systematic error that distorts the measured values but does not change the variance; ii) a systematic variance arising from a confounding factor that is either unknown or inadequately addressed; or iii) an interpretive error due to an inadequate or improper statistical method of analysis. One can also categorize these biases as biological, analytical, and interpretive according to whether their effects manifest in the biological experiment, the analytical measurements, or subsequent data analysis (Figure 1).

Of the biological biases, selection bias has historically been one of the most problematic for case-control experiments [5], which are very common in metabolomics observational studies. There are very many types of selection bias [5], but usually they represent an unbalanced selection of subjects that differ genetically or epigenetically. Also, temporal selection biases (when samples are taken) are especially hard to control for, given the natural cycles that exist in organisms. Biological conditions bias is another significant type of biological bias where some experimental condition endured by the subjects is not properly controlled for or considered.

Of the analytical biases, sample preparation bias usually presents the largest set of problems for the experimentalist. Any deviation in how and how long a sample is extracted, quenched, and stored can greatly impact the measured analytes [8, 9]. Standards bias represents the effects that different standards have on deriving some absolute or even relative quantification from a measurement. Sample complexity bias represents the effects on measurement due to physical interaction between a mixture of analytes present in a sample. Analytical conditions bias is due to some change in analytical conditions either during or between measurements.

Various interpretive biases and errors can also cause serious problems. However, many of the biases are simply born out of ignorance or laziness. Researchers tend to use the methods that they know and are easy to use, whether or not such methods are appropriate for the analysis. As scientists, it is a tendency that we should resist. Of the methodological biases, statistical assumptions about metabolomics data are most rampant. The two worst assumptions are that: i) nonsystematic error is completely Gaussian distributed and ii) measured variables are uncorrelated. The first assumption should never be made with analytical techniques like mass spectrometry, which by their very nature should include Poisson distributed error [10]. For NMR data, the nonsystematic error is often complicated and may include Lorentzian distributed observables, for which the variance is not analytically defined, but can be numerically estimated [11]. The second assumption of independence is unfounded given the correlation of metabolites in cellular metabolic networks. Another common interpretative bias is a lack of statistical power due to poor design and insufficient subject numbers. This situation is often confounded by a lack of correction for multiple testing. Also, assignment errors like the misassignment of a subject to a specific group (i.e. class assignment error or class noise) or the misidentification of an analyte can lead to serious misinterpretations. Another common interpretive bias is the use of preconceptions in interpretation, which can lead to a confirmation bias. For example, use of unverified metabolic models (i.e. a model of part or all of the metabolism of one or more specific organisms) is a common confirmation bias in metabolic data analysis. Also, the careless use of priors and limitation to expected metabolic models can lead to confirmation bias within a Bayesian statistical formalism [12].

### Dealing with biases

Constant vigilance is required to mitigate the many biases (Figure 1) that may add both significant systematic error and confounding systematic variance to a metabolomics dataset [6, 7, 13]. While there is no simple checklist of biases to look for and procedures to follow, there are several straight-forward strategies for dealing with many of these biases. The first is to use reasonably consistent experimental designs that exclude: i) partial consistencies for specific groups of samples, which may lead to a systematic variance from biological or analytical biases, and ii) trivial consistencies that may limit the generalization of results, due to systematic errors from biological or analytical biases. Building on the first strategy, the second strategy is to use effective experimental designs like matched-pair case-control experiments to limit the effects of confounding factors, especially from large biological biases. Also, the samples should be balanced between case-control groups for possible confounding factors (sex, age, related biological condition), in order to prevent systematic variance. The optimal design is equally balanced sampling (i.e. blocking) between the most likely confounding factors within case-control groups. Blocking allows the use of more sophisticated statistical methods [13]: i.e. ANOVA instead of a t-test or Welch ANOVA [14] instead of a Welch's t test [15].

The third strategy is to directly test how well a set of measured values for a given measured variable fits an expected/assumed analytical nonsystematic error distribution. Various implemented algorithms exist for testing how well a set of measured values fits different common distributions. For example, the nortest R package [16] includes several normality (normal distribution) tests including the Shapiro-Wilk test [17] and the Anderson-Darling test [18].

The fourth strategy is to validate results with temporally-separated datasets (i.e. analytical cross-validation) as a method for detecting the presence of biases. However, failure to detect bias by this method does not guarantee a bias-free approach.

The fifth strategy is to use blinded metabolomics experiments to reduce bias [6]. The double-blind randomized control trial is considered the gold standard for reducing researcher-introduced performance bias that can affect both biological and analytical conditions [19]. However, even this very effective method has known masking biases due to the psychological effects of the trial itself, especially when human subjects are involved [20]. Also, many observational studies cannot be double-blinded. Despite this, the blinding of analytical and/or statistical researchers (i.e. analytical and/or interpretive single-blinding) can reduce performance biases that affect both the analytical conditions and statistical interpretation [21].

The sixth strategy is to use analytical controls to correct for or to prevent analytical biases. However, application of this strategy is often specific to the analytical technique and instrumentation. With each sample in its own tube or vial, periodic controls or time-stamped near-random controls (random except to prevent neighbor effects) can be used to track analytical conditions during measurement [9]. For samples handled on plates, Latin square or 2D near-random patterns can be used [9]. An easier alternative representing a combination of strategies five and six involves the use of blind controls to detect and correct for several analytical biases including performance bias [22]. For full thoroughness, often a series of controls composed of complex mixtures of representative or chemically similar metabolites must be used to determine systematic error arising from sample extraction methods [23] and even from interactions between the metabolites themselves with respect to the specific analytical techniques employed. The latter is particularly problematic for electrospray mass spectrometry.

The seventh and final strategy is to fully document: i) the biological and analytical experimental procedures that produced a given metabolomics dataset; ii) the statistical procedures used in the analysis of the dataset; iii) a detailed list of all known or potential biases and assumptions, along with results of any analysis and testing of these bias and assumptions; and iv) results with adequate measures of uncertainty and confidence or at least a good explanation for why uncertainty and confidence measures are not provided. The two most common explanations are that not enough replicates were collected for error analysis or methods to determine the propagation of uncertainty do not exist for a specific statistical method or procedure. Such documentation enables thorough evaluation and peer-review by others and it facilitates future meta-analyses, especially when the datasets and documentation are deposited into public repositories like MetaboLights [24]. Minimum reporting standards for metabolomics experiments already exist for biological (plant specific) experimental procedures [25], analytical experimental procedures [26], and statistical procedures for analysis [27]. However, it would be better to augment these standards, especially for reporting known and potential sources of bias by borrowing from well-documented clinical standards [6] like STARD [28] and CONSORT [29, 30].

The overall purpose of these seven strategies is to identify which possible biases (Figure 1) may affect the proper interpretation of a metabolic experiment and then to limit their effect. However, in the end, a judgement call must be made on whether these strategies were adequate for the proper interpretation of the obtained metabolomics dataset(s).

## Methodology

### Standard error analysis methods

One central question that error analysis must address is whether nonsystematic error, variances, or covariances from either an analytical or biological source will prevent the desired detection and interpretation of biological systematic variance in a given metabolomics dataset. From this perspective, any nonsystematic error, variance, and covariance that could interfere with biological interpretation represent uncertainty that should be determined/estimated and properly addressed. To be most effective, error analysis needs to be part of the experimental design before the experiment even begins. Decisions must be made on the number of replicates at each stage of the experimental protocol in order to have the necessary dataset for thorough error analysis [31]. In some instances, it will be practically impossible to obtain the necessary replicates for a thorough error analysis and other approaches for estimating sources of variance and error will be necessary. Also, issues of statistical power must be considered for the statistical methods employed [34]. However, even when issues of statistical power are satisfied, typically when statistical power ≥ 0.8 at α=0.05 with “reasonable” statistical assumptions, it is advisable to test these “reasonable” assumptions by increasing analytical replicates for a subset of the samples. If these assumptions do not hold true or there is a lack of statistical power due to large analytical variance, then additional steps may be taken to address these issues, including: i) increasing the number of analytical replicates to deal with analytical nonsystematic error; ii) correcting for factors that cause analytical systematic variance; iii) switching to appropriate statistical methods that do not rely on these failed assumption(s); and/or iv) incorporating estimates of analytical variance and covariance into more sophisticated statistical methods.

So standard error analysis can be broken down into two major steps: i) error estimation and probability distribution testing and ii) error (uncertainty) propagation analysis. The first step involves the distribution testing, calculation/estimation, modeling, and comparison of nonsystematic error, variance, and covariance arising from biological and analytical sources. Testing for a normal distribution (normality test) is by far the most important probability distribution test. For normality testing, 8 to 10 replicates are considered the minimum needed with the Shapiro-Wilks test [35]. However, 20 to 30 replicates are typically desired for significant power, even though the Shapiro-Wilks test is considered the most statistically powerful normality test [17, 36]. When a measured variable fails normality tests, then other non-parametric or more fault-tolerant statistical methods should be employed to compensate for the lack of normality. For example, the non-parametric Wilcoxon-Mann-Whitney test is preferred to a t-test when the data are significantly non-normal [37–39]; but neither test works well if the data are highly skewed [40]. Sometimes, it is advantageous to interpret continuous measured variables categorically as discreet ranges and use binomial or multinomial statistical tests.

While the use of 3 analytical replicates is the minimum needed for quality control that includes the ability to detect outliers [9], 13 replicates (12 + 1) are considered the minimum for calculating variances with ∼half-width confidence intervals at least at the 90% confidence level when approximately normally distributed; 30 replicates are required to calculate variances with ∼half-width confidence intervals at the 99% confidence level [41]. In addition, these analytical replicates will naturally lower the standard error of the measured variable; however, there is a practical limit due to the availability of analytical instrumentation or of usable sample. Also, some analytical techniques have rather complex nonsystematic error structures that may pose additional difficulties [10, 42]. In these instances, a bootstrap procedure can be used to calculate confidence intervals [43, 44]. If the appropriate replicates across an experimental protocol are obtainable, then advanced mixed effect modeling methods can be employed to tease apart different sources of variance, including from random sources [32, 33, 45]. However, inadequate number of replicates requires the use of other approaches for estimating variance and its sources: i.e., estimation of variance indirectly from similar experiments, assuming biological variance is any variance not directly attributable to an analytical source, and/or modeling variance from time series measurements [46].

1 $$y=f(x_{1},\ldots ,x_{n})\approx \underset{\text{0th order}}{\underset{︸}{f({\bar{x}}_{1},\ldots ,{\bar{x}}_{n})}}+\underset{\text{1th order}}{\underset{︸}{\sum \frac{\partial f({\bar{x}}_{1},\ldots ,{\bar{x}}_{n})}{\partial x_{i}}(x_{i}-{\bar{x}}_{i})}}[\underset{\text{2th order}}{\underset{︸}{+\frac{1}{2!}\sum \sum \frac{\partial^{2}f({\bar{x}}_{1},\ldots ,{\bar{x}}_{n})}{\partial x_{i}\partial x_{j}}(x_{i}-{\bar{x}}_{i})(x_{j}-{\bar{x}}_{j})+\ldots }}$$

2 $$\bar{y}=\frac{1}{N}\sum_{a}^{N}f(x_{1,a},\ldots ,x_{n,a})\approx \frac{1}{N}\sum_{a}^{N}\left(f({\bar{x}}_{1},\ldots ,{\bar{x}}_{n})+\sum \frac{\partial f({\bar{x}}_{1},\ldots ,{\bar{x}}_{n})}{\partial x_{i}}(x_{i,a}-{\bar{x}}_{i})\right)\approx f({\bar{x}}_{1},\ldots ,{\bar{x}}_{n})+\sum \frac{\partial f({\bar{x}}_{1},\ldots ,{\bar{x}}_{n})}{\partial x_{i}}\frac{1}{N}\sum_{a}^{N}f(x_{i,a},\ldots ,{\bar{x}}_{i})\approx f({\bar{x}}_{1},\ldots ,{\bar{x}}_{n})+\sum \frac{\partial f({\bar{x}}_{1},\ldots ,{\bar{x}}_{n})}{\partial x_{i}}(0)\approx f({\bar{x}}_{1},\ldots ,{\bar{x}}_{n})$$

3 $$\begin{array}{c} \sigma_{y}^{2}=\frac{1}{N-1}\sum_{a}^{N}{\left(y_{a}-\bar{y}\right)}^{2}\\ =\frac{1}{N-1}\sum_{a}^{N}{\left(f(x_{1,a},\ldots ,x_{n,a})-f\left({\bar{x}}_{1},\cdots ,{\bar{x}}_{n}\right)\right)}^{2}\\ \approx \frac{1}{N-1}{\sum_{a}^{N}\left(f\left({\bar{x}}_{1},\cdots ,{\bar{x}}_{n}\right)+\sum \frac{\partial f\left({\bar{x}}_{1},\cdots ,{\bar{x}}_{n}\right)}{\partial x_{i}}(x_{i,a}-{\bar{x}}_{i})-f\left({\bar{x}}_{1},\cdots ,{\bar{x}}_{n}\right)\right)}^{2}\\ \approx \frac{1}{N-1}{\sum_{a}^{N}\left(\sum \frac{\partial f\left({\bar{x}}_{1},\cdots ,{\bar{x}}_{n}\right)}{\partial x_{i}}(x_{i,a}-{\bar{x}}_{i})\right)}^{2}\\ \approx \frac{1}{N-1}\sum_{a}^{N}\left({\sum (\frac{\partial f\left({\bar{x}}_{1},\cdots ,{\bar{x}}_{n}\right)}{\partial x_{i}}(x_{i,a}-{\bar{x}}_{i}))}^{2}+\sum \sum_{j\neq i}\frac{\partial f\left({\bar{x}}_{1},\cdots ,{\bar{x}}_{n}\right)}{\partial x_{i}}\frac{\partial f\left({\bar{x}}_{1},\cdots ,{\bar{x}}_{n}\right)}{\partial x_{j}}\left(x_{i,a}-{\bar{x}}_{i}\right)\left(x_{j,a}-{\bar{x}}_{j}\right)\right)\\ \approx \underset{\text{variance sum}}{\underset{︸}{\sum {\left(\frac{\partial f\left({\bar{x}}_{1},\cdots ,{\bar{x}}_{n}\right)}{\partial x_{i}}\right)}^{2}\sigma_{x_{i}}^{2}}}+\underset{\text{covariance sum}}{\underset{︸}{\sum \sum_{j\neq i}\frac{\partial f\left({\bar{x}}_{1},\cdots ,{\bar{x}}_{n}\right)}{\partial x_{i}}\frac{\partial f\left({\bar{x}}_{1},\cdots ,{\bar{x}}_{n}\right)}{\partial x_{j}}r_{x_{i}x_{j}}}}\sigma_{x_{i}}^{2}\sigma_{x_{j}}^{2} \end{array}$$

4 $$\sigma_{y}^{2}\approx j{\left(\bar{\text{x}}\right)}^{T}C_{x}j(\bar{x})$$

5 $$C_{y}\approx J_{f}(\bar{x})C_{x}J_{f}{\left(\bar{x}\right)}^{T}$$

Calculating or estimating covariance is typically problematic for metabolomics datasets due to the small number of replicates *n* versus the number of measured variables *p* (*n*<<*p*). Also, reasonable guidelines for calculating/estimating covariances are not straight-forward and the number of analytical replicates needed often depends on the number of measured variables and the amount of correlation to accurately detect and estimate. One rule of thumb based on single correlation estimation is *n* ≈ 16/(ln((1+*r*)/(1-*r*)))^2^ for detecting a correlation *r* at an α (Type I error) of 0.05 and power of 80%, but this rule ignores the fact that *p*(*p*-1)/2 correlations are being estimated [41]. Despite these problems, there are ways to deal with the *n*<<*p* condition in metabolomics datasets: i) by using high variance of covariance to weight towards an expected covariance matrix structure [47]; ii) by averaging analytical covariance across biological replicates [31]; and iii) by using known variance-covariance relationships to estimate an analytical covariance matrix from calculated analytical variances [48].

### Error (uncertainty) propagation analysis

The propagation of uncertainty (error) through functions and algorithms is analyzed by two fundamentally different types of methods: i) mathematical (analytical) derivation and approximation and ii) numerical analysis. Several software platforms exist to facilitate the use of these methods [49]; however, custom implementation of these methods is often necessary to handle specific issues of a given metabolomics dataset and its analysis. With few exceptions, almost all analyses of error propagation via mathematical derivation and approximation are performed from a linear perspective [50]. This linear assumption is used, whether the functions and algorithms being analyzed are linear or nonlinear [1]. To fully understand the effects of this linear perspective, we start with Equation 1, which describes the multivariate Taylor series approximation for a given function *f*. Most error propagation analyses use only the 0^th^ and 1^st^ order terms, due to the exponential growth of higher order terms, as highlighted by the sum of sums of 2^nd^ order terms versus the simple sum of 1^st^ order terms in Equation 1. However for linear equations, this approach simplifies to an exact solution, since all 2^nd^ order and higher terms are zero.

In Equation 2, the approximation of $\bar{y}$ (mean of *f*), using only the 0^th^ and 1^st^ order terms of the series, simplifies to f applied to $\bar{x}$ (mean of vector **x**). This sets up for the approximation of $\sigma_{y}^{2}$ (variance of *f*), again using only the 0^th^ and 1^st^ order terms of the series (Equation 3). The rearrangement of terms simplifies to sums of variance and covariance terms that approximate the effects of the variances of **x** on *f* using only the tangent of *f* at $\bar{x}(\partial f/\partial x_{1}({\bar{x}}_{1},\ldots ,{\bar{x}}_{n}))$. Thus, the sums of variance and covariance terms are just linear approximations for the propagation of uncertainty through *f*. These sums of variance and covariance terms are often represented with a simpler vector/matrix notation (Equation 4), where **C**_**x**_ is the covariance matrix for **x** (sometimes called a variance-covariance matrix and represented as **S**_**x**_). And **j(** $\bar{x}$ **)** is the vector of partial derivatives at $\bar{x}$, i.e. $\left[\partial f/\partial x_{1}(\bar{x}),\ldots ,\partial f/\partial x_{n}(\bar{x})\right]$ [51, 52]. This concept can be further generalized for a system of equations **y** = *F*(**x**) as shown in Equation 5, allowing the calculation of **C**_**y**_ using the Jacobian matrix of all first-order partial derivatives of *F* at $\bar{x}$ **, i.e. J**_*F*_($\bar{x}$). But when no correlation exists between *x*_*i*_ variables, Equation 3 simplifies to just a sum of variance terms (or standard error terms) [2, 53], which is often referred to as Gaussian error propagation (GEP). It is this “no correlation” GEP version that underlies almost all standard error propagation rules [1]. However, this GEP approximation can be very poor when significant correlation exists and/or for nonlinear functions when $\bar{x}$ is near a critical point or an inflection point [50].

### Numerical error propagation analysis

Owing to these approximation problems with nonlinear functions and complex algorithms or significant deviations from normality, a second approach to error propagation using numerical analysis is often more accurate and much easier to implement in these situations, which are typical for metabolomics data analysis. The most common numerical approach is known as the Monte Carlo method, which samples a given function or algorithm applied to random input values [54]. However, from this broad definition, the Monte Carlo method is really a large collection of methods with a wide variety of applications beyond the scope of this review [55]. Therefore, we will focus on a simple Monte Carlo method (Equation 6) where a set of pseudo-random input vectors of values (**x**_i_ ∈ *X*) with specific probability distributions (*X*_*j*_ ∼*D*_*j*_) are used to generate a set of vectors of values (**y**_i_ ∈ Y) from a given function or algorithm (*f*) for analyzing the propagation of uncertainty through *f*. However, there are more advanced Monte Carlo approaches that can analyze the propagation of uncertainty from both repeated measurements and other information within a Bayesian framework [56]. As previously mentioned, it is important to characterize the probability distributions for the vector of input variables arising from analytical procedures via testing of common probability distributions and the calculation or estimation of expected values, variances, and correlations from experimental data.

6 $$y_{i}=f(x_{i})\;\text{where}\;x_{\text{i}}\in X\text{and}X_{j}\sim D_{j}$$

With these statistical characteristics, there are several ways to generate a pseudo-random sampling for the input variables for common probability distributions. Both Matlab [57] and R (a free, open source, statistical programming language that is robustly supported) [58] have built-in functions for generating pseudo-random values for most of the common probability distributions There are also straight-forward algorithms to calculate a set of pseudo-random values that fit several of the common probability distributions using a set of uniformly distributed pseudo-random values (i.e. U[0,1]) [59, 60]. In particular, the Box Muller method is a popular (easy to implement) algorithm for calculating normally distributed pseudo-random pairs of values from pairs which are U[0,1] distributed [60]. Also by definition, the inverse of a cumulative distribution function can be used to calculated pseudo-random values from U[0,1] distributed values [61]. But care should be taken in selecting a good uniformly distributed random number generator for use in generating a given distribution. An enhanced Wichmann-Hill algorithm is recommended by the current draft of the Guide to the Expression of Uncertainty in Measurements [62], but other algorithms have passed rigorous testing as well [63].

Even pseudo-random values of complex or unknown distributions can be estimated using a two-sample Kolmogorov–Smirnov test (K-S test), which is a non-parametric test that compares the distributions of two samples and determines whether they deviate significantly or not [64, 65]. In this simple approach, sets of pseudo-random values are generated based on bootstrap-derived statistical parameters and tested against an experimentally derived set of measured values using the two-sample K-S test. But there are also various approaches to simulate significantly non-normal multivariate random variables that even include correlation [66, 67] or approaches that take random variables with given distributions and introduce correlation [68]. Finally, with the appropriate set of pseudo-random input vectors of values (**x**_i_ ∈ *X*), application of the given function or algorithm (Equation 6) produces a set of vectors of values (**y**_i_ ∈ Y) that can be directly analyzed in an analogous manner as experimental data, i.e. probability distribution testing and calculation of expected values, variances, standard errors, and correlations when common probability distributions are present.

If the function *f* is linear and *X* variables are (reasonably) independent and identically distributed (*X*_*i*_ ∼ *X*_*j*_) with finite variance, then *Y* often reflects the distribution of *X* (*Y*_*i*_ ∼ *X*_*j*_) or even approximates a normal distribution for *Y* variables (*Y*_*i*_) that depend on many *X* variables (*X*_*j*_). However, if *f* is nonlinear, then drastically non-normal distributions are common for *Y* and quite distinct from *X*, even if *X* is normally distributed. And nonlinearity is very common for metabolic models with exchange and bidirectional fluxes, which sometimes can be solved by a linearization of the model [69]. However, in non-linearizable situations or simply when the *Y* variables are very non-normal (a multimodal distribution for example), a median with a confidence interval is preferable to a mean with a standard error, and can be estimated by a straight-forward approach with samples sizes of 1000 or 10000, depending on the desired level of confidence in these confidence intervals [70]. The method simply orders the sampling for each *Y*_*i*_ and takes the interval (*y* ^*(n+1)(1-c)*^ *, y* ^*(n+1)c*^) where c is the level of confidence as a fraction (for example 0.95). Likewise, a Spearman's rank correlation coefficient can be used to calculate correlation in a non-parametric way in these situations [71].

### Error propagation in inverse problems

Another common numerical approach used to analyze metabolomics data is the optimization of parameters in an inverse problem [72]. Often a model (*g* in Equation 7) with parameters (**y**_i_) for calculating values (**x**_i_) that are directly comparable to experimental data (**x**_*exp*_) is much easier to construct than an analytical function (*f* in Equation 6) that can calculate desired parameters from experimental data, especially when experimental data is collected in a time series [73]. For example, a model of the relevant chemical reactions for a “known” cellular metabolic network (to the limited degree of our current scientific knowledge) is more easily constructed and used to calculate specific metabolite fluxes and pools (mass-related characteristics) that can be compared to experimental values [74–76]. These models are becoming more available and easier to use and modify thanks to databases like Biomodels.net and associated modeling tools [77, 78]. However, care must be taken in the interpretation of the term “model”: sometimes model refers to just the framework of equations (*g)*, sometimes it refers to g and fixed input parameters (*y*_*i,j*_ = c_*j*_), and sometimes it refers to g and optimized parameters (**y**_*opt*_). For the rest of this discourse, model will simply refer to *g*.

7 $$x_{i}=g(y_{i})\text{where}g\approx f^{-1}$$

Equation 8 describes an objective function (*O*_*s*_ - simple), also known as a target function or energy function depending on context, which compares the results from this model **x**_*i*_ with experimental data **x**_*exp*_ through some norm function, like an $\ell_{2}$-norm in this instance. This objective function is minimized while model parameters are optimized to **y**_*opt*_ using an optimization method of choice, which is often some type of Monte Carlo method by definition (cf. simulated annealing).

8 $$O_{s}(y_{i})={\left\|g(y_{i})-x_{\text{exp}}\right\|}^{2}={\left\|x_{i}-x_{\text{exp}}\right\|}^{2}={\sum_{j}\left(x_{i,j}-x_{\text{exp},j}\right)}^{2}$$

However, almost all metabolomics inverse problems are ill-posed due to model complexity and non-linearity, limitations in the number and variety of measurements, and significant amounts of uncertainty in the measurements. These characteristics give rise to common properties of ill-posed problems: i) preclusion of a unique solution **y**_opt_ to a given set of experimental measurements **x**_*exp*_; ii) the existence of multiple solutions **y**_opt,I_; iii) the presence of discontinuities in the objective function; and iv) high conditioning (i.e. large variation) in model parameters with respect to small changes in experimental measurements. Regularization of the ill-posed objective function as a better-posed objective function is required to mitigate these properties of ill-posedness and to minimize the amplification of analytical uncertainty in the derived model parameters **y**_*opt*_ during optimization. Without such regularization, the optimization of the objective function in Equation 8 quickly leads to overfitting of model parameters to the error in the experimental data. However, an important question with regularization is how much information is present in the available data, and to what extent prior knowledge can be used to overcome limitations in the data without introducing undue bias?

Tikhonov regularization is a common regularization method used to prevent overfitting in inverse problems, due to its effectiveness and simplicity of implementation [79]. Tikhonov regularization uses *a priori* knowledge of expected model parameters (**y**_*E*_) to allow stable optimization with the experimental data (**x**_*exp*_) while minimizing fitting to its error [80]. Equation 9 incorporates a Tikhonov regularization *R*(**y**_*i*,_ **y**_*E*_) as a *p*-norm (∣∣**w**∣∣_p_) into Equation 8 with a certain weighting α, which must be kept small enough to prevent any significant bias to **y**_*E*_ but large enough to prevent overfitting [79], and a *p* that properly balances between the norms ∣∣**x**_i_∣∣ and ∣∣**y**_i_∣∣ [81]. Once the issues of picking a proper α and p are overcome, even a confidence region around **y**_*E*_ that includes **y**_*exact*_ can be estimated with respect to ${\left\|y_{i}-y_{E}\right\|}_{p}^{2}$ based on a Fisher distribution, if normality conditions hold and the dimensionality of **x**_exp_ is significantly greater than the dimensionality of **y**_*E*_ [72, 82].

9 $$O_{s}(y_{i})={\left\|g(y_{i})-x_{\text{exp}}\right\|}^{2}+\alpha R(y_{i},y_{E})={\left\|x_{i}-x_{\text{exp}}\right\|}^{2}+\alpha {\left\|y_{i}-y_{E}\right\|}_{p}^{2}={\sum_{j}\left(x_{i,j}-x_{\text{exp},j}\right)}^{2}\alpha {\left(\sum_{k}{|y_{i,k}-y_{E,k}|}^{p}\right)}^{\frac{2}{p}}$$

But when the analytical error for **x**_*exp*_ can be determined, a more general approach for regularization can be employed, where the optimization is stopped when the objective function is below the observed analytical error [72]. One error-bounded generalized least squares implementation, shown in Equation 10, stops optimization when the objective function (*O*_*g*_) is below an error threshold δ_*x*_. This threshold can be approximated by a χ^2^ statistic $\chi_{n-m}^{2}(1-\beta )$, with *n-m* degrees of freedom and a p-value of -β, where n is the number of measured experimental variables, m is the number of parameters in the model, and β is the desired level of confidence. However, this implementation assumes that *O*_*g*_(y_opt_) < $\chi_{n-m}^{2}(1-\beta )$, where y_opt_ is determined by the lowest *O*_*g*_(y_i_). With this assumption holding true, it is straight-forward to estimate the confidence region *CR*_1*-*β_(y_opt_) and the individual confidence interval *CI*_*yj,I-β*_(y_opt_) with a large set of optimizations in Equations 11 and 12 respectively [83].

10 $$O_{g}(y_{i})={\left(g(y_{i}))-x_{\text{exp}}\right)}^{T}C_{x}^{-1}(g(y_{i})-x_{\text{exp}})\leq \delta_{x}$$

11 $$CR_{1-\beta }(y_{opt})\approx \left\{y_{i}|O_{g}(y_{i})\leq \delta_{x}\approx O_{g}(y_{opt})+\chi_{n-m}^{2}(1-\beta )\right\}$$

12 $$CI_{y_{j},1-\beta }(y_{opt})\approx \left\{y_{j0}|\min O_{g}(y_{i}){|}_{y_{j}=y_{j0}}\leq \delta_{x}\approx O_{g}(y_{opt})+\chi_{1}^{2}(1-\beta )\right\}$$

However, this general approach in Equations 10, 11, and 12 only holds if all of the measured variables are normally distributed and the analytical covariance matrix **C**_**x**_ is known or well-estimated. Also for Equation 11, the residuals normalized by the square root of the covariance matrix (i.e. normalization matrix $C_{x}^{-1/2}$ should be tested for normality. Plus, the optimization needs a large number of repetitions; however, the extra computational requirements can sometimes be mitigated by optimization methods that take advantage of first and second order partial derivatives, typically in the form of Jacobian and Hessian matrices [83]. If the measured experimental variables are not normally distributed, then it is extremely challenging to devise an objective function that will properly weight the residuals to a single error threshold and preserve the underlying distributions and covariance structure. There is another Monte Carlo method that estimates the confidence region and confidence interval via an analytical estimation of the covariance matrix of **y** (**C**_**y**_) from the inverse of the Jacobian matrix of g normalized by the square root of the analytical covariance matrix, [$C_{x}^{-1/2}$ **J**_*g*_]^-1^ [69]. However, it suffers from both the need for normally distributed measured variables and the linear approximations made in the estimation.

### Metabolic model verification and selection

Equations 8 through 12 are based on the grand assumption that model *g* is “reasonably” accurate, which has the potential of being a very large interpretive bias. Moreover, the faith in certain metabolic models is quite troubling, given the lack of verified details and errors in metabolic databases used in the construction of models of metabolic networks, especially models of eukaryotic metabolic networks [84]. Also, many metabolic models include more parameters than measured variables, which greatly limits the ability to verify such models. Given these caveats, there are two general approaches for improving model verification: a) pare down the metabolic model to what is relevant to the observables; and b) design experiments where there are enough observables to perform model selection (i.e. *n* >> *m*).

For the first approach, there are three main ways to pare down a metabolic model: i) gross model paring, ii) specific variable pairing by independence, and iii) specific variable paring by sensitivity. Gross model paring is simply limiting the model to relevant pathways and modules of a metabolic network [85], assuming that they are known. Specific variable pairing by independence limits the model parameters to the smallest set of independent or “free” model parameters from which other intermediate model parameters are derived [86]. An optimal set of free model parameters can be directly calculated via the determination of a basic set for the null space of the stoichiometry matrix [83]. Specific variable paring by sensitivity removes and/or simplifies parts of a model that include insensitive model parameters with respect to measured experimental variables. Determining which model parameters are insensitive to measured experimental variables can be done in a directed manner by carefully removing parts of a model and seeing if this model change does not appreciably worsens the objective function nor appreciably change the optimized values of other model parameters [87]. Also, the relative size of model parameter confidence intervals can help direct this paring. But more sophisticated analyses require calculating a sensitivity matrix (i.e. Jacobian matrix of *f*, **J**_*f*_), which is only straight-forward when the direct model *f* (from Equation 6) can be derived [88]. However, an estimation of the model parameter sensitivity matrix can be calculated, starting with an inverse of the Jacobian matrix of *g* normalized by the square root of the analytical covariance matrix, [$C_{x}^{-1/2}$ **J**_*g*_]^-1^ in a few steps [69, 83]. Both of these analytical approaches represent a linear interpretation of sensitivity with limitations similar to analytical error propagation as described above.

The second approach for improving metabolic model verification is to design experiments where there are significantly more measurables than model parameters. For metabolomics experiments, this has been greatly aided by the combined use of stable isotopes along with analytical techniques like NMR and ultra-high resolution mass spectrometry that can detect the specific incorporation of these labeling isotopes in metabolites [89–92]. The ability to detect specific isotopomers by NMR and isotopologues (mass-equivalent sets of isotopomers) by ultra-high resolution MS, greatly increases the number of possible measured experimental variables and provides a rigorous internal normalization that obviates the need for external controls, when absolute quantification is not necessary. In addition, there are several potential multiplicative factors on the number of measurable like time series measurements, the use of multiple stable isotopes (^13^C, ^15^N, ^2^H), and the use of multiple isotope labeling source metabolites. In fact, the design of metabolomics is coming full circle, where metabolic models are being used to design optimal stable isotope labeling experiments [93–96]. However, this approach for metabolomics experimental design appears more robust for high quality metabolic models from model prokaryotic organisms.

Once appropriate metabolic models are constructed, model parameters pared, and enough experimental data collected (n >> m), models should be verified to limit significant interpretive confirmation bias. Often this verification process starts with gross measurement error detection via analysis of elemental and heat balances through the metabolic stoichiometry matrix, if the appropriate measurements are available [97, 98]. If analytical error of the experimental data can be adequately determined or estimated and the measured variables are approximately normally distributed, then the model can be further verified by a goodness-of-fit test based on a χ^2^ statistic that reflects the number of degrees of freedom in the optimization and a desired level of confidence. When *O*_*g*_(**y**_opt_) > $\chi_{n-m}^{2}(1-\beta )$ (see Equation 10), the model should be rejected, especially if gross measurement errors can be ruled out [83]. However, rejection of a model is just a starting point for its improvement [99] and parts of the model involving parameters which are highly sensitive to small changes in measured variables are often a good place to look [83]. Eventually selection of models using standard methods like Akaike information criterion [100] should be a goal of the field, but these methods require approaches that limit the effects of overfitting during model parameter optimization, like the use of independent sets of measurements [101].

## Discussion

Metabolomics has a range of applications including: the discovery or detection of biomarkers related to a cellular, physiological, or disease state of interest; the generation and verification of biochemical mechanism-based hypotheses for biological processes and phenomena; and the improvement of industrial fermentation processes via simulation. In all of these applications, the accurate determination of how uncertainty propagates through data analysis is required for the proper evaluation of results. Towards this end, experiments should be designed to minimize both biological and analytical error, to have the appropriate statistical power and controls, and to contain enough replicates to derive the analytical error and covariance, for at least a portion of the biological samples. Also, data and error analyses should include the testing of statistical assumptions which drive the use of appropriate statistical methods. One excellent example that embodies these principles involves the use of an analytical correlation matrix estimated from analytical replicates and used in a maximum likelihood principal component analysis instead of a standard principal component analysis [31, 48].

In addition, the reporting of results in journals and data repositories must accurately include the derived and propagated uncertainty in the results. All p-values must be properly or at least conservatively adjusted under multiple testing conditions to prevent over-optimistic interpretation, especially in biomedical settings [7]. Also, p-values should be included with correlations (R^2^) derived from regression analysis. Standard errors and confidence intervals should be reported for all measured and derived variables, when reasonably possible. The lack of reporting confidence region/intervals for published metabolic model parameters is due to a host of factors including: the requirement for approximately normally distributed measured variables, a reasonable metabolic model, requirement for well-estimated analytical covariances, requirement for more measurements than model parameters, error analysis expertise, and computational expense. But a few examples of reporting do exist ([102] for example), which will hopefully become the norm in the future. Moreover, a focus on error analysis in the development of and documentation of data analysis methods is critical to changing reporting habits.
